# Phenotypic and whole genome characterization of multidrug-resistant *Campylobacter coli* from chicken liver

**DOI:** 10.1016/j.psj.2026.107271

**Published:** 2026-06-10

**Authors:** Mª Pilar González-Navarro, Alicia Manzanares-Pedrosa, Florencia Correa-Fiz, Lauge Holm Sørensen, Teresa Ayats, Núria Aloy, Miquel Nofrarías, Rene S. Hendriksen, Marta Cerdà-Cuéllar

**Affiliations:** a Unitat mixta d’Investigació IRTA-UAB en Sanitat Animal, Centre de Recerca en Sanitat Animal (CReSA), Campus de la Universitat Autònoma de Barcelona (UAB), Bellaterra 08193, Catalonia, Spain; b IRTA, Programa de Sanitat Animal. Centre de Recerca en Sanitat Animal (CReSA), Campus de la Universitat Autònoma de Barcelona (UAB), Bellaterra 08193, Catalonia, Spain; c Technical University of Denmark (DTU), National Food Institute. Lyngby, Lyngby-Taarbæk Municipality, Copenhagen, Denmark

**Keywords:** Antimicrobial resistance, *Campylobacter*, Broiler chicken, Liver, WGS

## Abstract

Campilobacteriosis is the most frequently reported zoonosis in the European Union. Poultry and poultry products are the primary sources of human infection. The high occurrence of antibiotic-resistant (AR) *Campylobacter* strains is particularly concerning, as it complicates treatment and poses a serious public health threat. Therefore, this study assessed the prevalence of AR *C. coli* recovered from the gut and liver of broiler chickens from northeastern Spain. Using the broth microdilution method and whole genome sequencing (WGS) with Illumina HiSeq, the AR of 40 *C. coli* strains (n = 18 from liver; n = 22 from gut) was assessed. Phenotypically, all isolates were resistant to ciprofloxacin (100%), followed by tetracycline (95%), while co-resistance to both constituted the most prevalent profile (25%). Moderate to high resistance frequencies were observed to erythromycin (53%), ertapenem (28%) and gentamicin (23%). Overall, 70% of the isolates showed a multidrug resistant (MDR) phenotype. The origin of the strain (caeca or liver) did not influence the occurrence of AR. WGS revealed that all isolates harbored the *gyrA* T86I mutation conferring ciprofloxacin resistance while most tetracycline-resistant isolates (80%) carried the *tet*(*O*) gene. Macrolide-resistant isolates either exhibited a point mutation in the 23S rDNA or the 50S ribosomal L22 protein gene, or harbored *ermB* and *erm(53)* genes. *BlaOXA* genes were present in 72.5% of the ertapenem-resistant strains; however, ertapenem-specific resistance was not identified and might involve the CmeABC efflux pump with certain *blaOXA* genes. Among gentamicin resistant strains, 78% harbored the *aph(2″)* gene conferring resistance to this antibiotic. All isolates carried genes coding for the CmeABC and CmeDEF efflux pumps conferring unspecific AR. Overall, a strong concordance between phenotypes and genotypes was seen, with WGS analysis also revealing additional putative resistance determinants and predicting 92.5% MDR strains. This study highlights the potential risk of chickens and chicken livers as a source of AR *C. coli* and emphasizes WGS as a valuable tool providing significant additional information on the AR potential of *C. coli* isolates.

## Introduction

*Campylobacter* is one of the main causes of human gastroenteritis worldwide (Havelaar et al., 2015; EFSA and ECDC, 2025a) Since 2005, campylobacteriosis has been the most reported zoonotic infection in the European Union (**EU**), surpassing salmonellosis and causing more than 168,396 human cases and around 96 million cases worldwide (EFSA and ECDC, 2025a). The most relevant species causing campylobacteriosis are thermophilic *Campylobacter jejuni* (*C. jejuni*) and *Campylobacter coli* (*C. coli*) which are also the most prevalent species found in broiler chickens (EFSA, 2010). Broiler chickens are the main source of *Campylobacter* infection although *Campylobacter* species are ubiquitous with a great diversity of hosts acting as asymptomatic carriers. It has been shown that thermophilic *Campylobacter* spp. in broilers can reach extraintestinal tissues and spread to edible organs such as the liver reaching up to 80% in prevalence (Jones et al., 2016; Prendergast et al., 2025). Since the liver is a product often served undercooked, it frequently fails to reach the recommended 70 °C internal temperature. At this temperature, the survival of *Campylobacter* has been reported to range from 48% to 98% (Jones et al., 2016), thereby increasing the risk of food-borne disease.

Campylobacteriosis is often self-limiting, however in severe cases, antibiotic treatment is needed. Macrolides and fluoroquinolones are the treatment of choice (Dai et al., 2020). Despite uncommon, long-term conditions can occur following campylobacteriosis, including reactive arthritis, irritable bowel disease, or neurological disorders such as Guillain-Barré syndrome and Miller Fisher syndrome (Connerton and Connerton, 2017).

Extensive and inappropriate use of antibiotics in human and veterinary medicine has led to the emergence of antibiotic resistant (**AR**) and multidrug resistant (**MDR**) *Campylobacter* strains (Signorini et al., 2018), limiting the treatment options of human campylobacteriosis. Hence, AR *Campylobacter* species are recognized as a major global health threat (Havelaar et al., 2015; WHO, 2019). MDR strains have increased among livestock populations, posing a serious public health concern (EFSA and ECDC, 2025b). *Campylobacter* spp. can carry a diversity of genetic determinants conferring antibiotic resistance, which can be acquired by spontaneous mutations, conjugation, transformation or horizontal gene transfer. Mechanisms of resistance include chromosomally or plasmid-encoded resistance genes (ARGs), such as those conferring resistance to tetracyclines (e.g. *Tet(O)*); point mutations, which confer resistance to macrolides and fluoroquinolones (e.g. *GyrA* gene p.T86I); with efflux pumps such as cmeABC and to a lower extent cmeDEF, contributing to the intrinsic resistance to a broad range of structurally unrelated antimicrobial agents by lowering the antibiotic accumulation in bacterial cells (Akiba et al., 2006; Wieczorek and Osek, 2013)

These resistance mechanisms often act synergistically, contributing to the emergence of MDR isolates (Wieczorek and Osek, 2013; Cobo-Díaz et al., 2021).

Although traditional methods to determine antimicrobial susceptibility have been widely used to characterize AR phenotypically, genomic methods are increasingly used due to their higher accuracy and discrimination power (Cantero et al., 2018). Whole genome sequencing (**WGS**) has proven to be an effective tool to reveal resistance mechanisms and therefore predict resistance phenotypes (Whitehouse et al., 2018), showing a high concordance between phenotypic AMR and genotype-predicted resistance (Cantero et al., 2018; Manzanares-Pedrosa et al., 2025a). Unlike phenotypic methods, WGS provides more accurate insights into AR mechanisms improving the understanding of AR evolution and epidemiology (Manzanares-Pedrosa et al., 2025a).

Given the limited information about AR of *C. coli* isolates from edible tissues, we aimed to assess the AR of *C. coli* isolates from broiler chickens and the genetic mechanisms conferring such AR through WGS.

## Materials and methods

### Campylobacter coli isolates

A total of 40 extraintestinal and intestinal strains from livers (n = 18) and the caecum (n = 22) were analyzed. They were obtained from carcasses collected at slaughter from commercial farms during two different sampling periods: cecal samples from 12 farms sampled from 2010 to 2013 (Sommer et al., 2016), and from ceca and livers from 21 farms sampled between 2019 and 2020 (Manzanares-Pedrosa et al., 2025a). At slaughter, caeca and livers were obtained during evisceration from randomly collected carcasses from different farms and flocks. Typically, one isolate per flock and type of sample was selected for the study. Strains were isolated by standard culture by direct plating onto blood-free selective agar (mCCDA, modified charcoal cefoperazone desoxycholate agar, CM739; with selective supplement SR0155E; Oxoid, Basingstoke, UK) as previously described (Manzanares-Pedrosa, et al., 2025a) and were identified at species level by multiplex PCR using primers targeting the lipid A (*lpxA*) gene (Klena et al., 2004). All strains were preserved at −75°C in brain-heart infusion broth containing 20% glycerol until used.

### Antibiotic susceptibility testing

The minimum inhibitory concentration (**MIC**)-based broth microdilution method with EUCAMP3 plates (Thermofisher Scientific, Spain) was used. Antibiotics tested were included in the EU monitoring program of AR for food-producing animals and meat derived thereof. The following antibiotics and ranges were tested: ciprofloxacin (**CIP**; 0,12-32 µg/ml), tetracycline (**TET**; 0.5-64 µg/ml), erythromycin (**ERY**; 1-51 µg/ml), ertapenem (**ETP**; 0.12 4µg/ml), gentamicin (**GEN**; 0.25-16 µg/ml) and chloramphenicol (**CHL**; 2 64 µg/ml). *C. jejuni* ATCC33650 was used as a control strain. Strains were categorized as susceptible or resistant according to the epidemiological cut-off values following the EUCAST guidelines (https://mic.eucast.org/search/; MIC distributions for *C. coli* September 2025). Strains were considered MDR when they showed resistance to three or more classes of antibiotics.

### WGS analysis

Genomic DNA was extracted using the Wizard Genomic DNA Purification Kit (Promega Corporation, Madison, Wisconsin, USA) according to the manufacturer’s instructions. All the strains were submitted to Novogene pair-end sequencing (Illumina HiSeq). The libraries were prepared with Nextera XT DNA sample preparation kit (cat. no. FC-131-1024; Illumina, Inc., San Diego, CA, USA) followed by multiplexed paired-end sequencing with a read length of 150 bp.

The raw sequences were processed by initial checking quality and trimming the adapters with FASTP v0.22.0 (Chen et al., 2018). Two *de novo* assembly tools, SPADES v3.15.5. (Bankevich et al., 2012) and UNICYCLER v0.5.0, (Wick et al., 2017) were tested on a subsample of reads. The assembly of all genomes was finally performed with SPADES v3.15.5 since it outputs 16% less number of contigs than UNICYCLER v0.5.0 while covering similar genome fraction. Afterwards, contig refinement was performed with CIRCLATOR v1.5.5 (Hunt et al., 2015) and QUAST was used to assess the quality of the assemblies (Gurevich et al., 2013). The assembled genomes obtained were analyzed with the CamPype workflow (Ortega-Sanz et al., 2023) to identify known ARGs and resistance-associated point mutations, providing a genomic profile of the potential AR for each strain. A mass screening of the contigs for ARGss was performed with ABRicate v1.0.1 (T. Seemann, https://github.com/tseemann/abricate) with multiple reference databases included, i.e. ARG-ANNOT (Gupta et al., 2014), CARD (McArthur et al., 2013), MEGARes (Lakin et al., 2017), NCBI and ResFinder (Zankari et al., 2012), all available through the Center for Genomic Epidemiology (https://cge.cbs.dtu.dk/services/ResFinder). Additionally, AMRFinderPlus v3.11.2 (Feldgarden et al., 2021) was used to identify both ARGs and resistance-associated chromosomal point mutations based on protein and nucleotide alignments against the NCBI Bacterial Antimicrobial Resistance Reference Gene Database (BioProject PRJNA1440180). The cut-off used in the different tools to decide whether a gene was present or not was as follows: any hit with coverage below 80% and identity below 60% was removed.

To investigate the presence of plasmids, the assembled contigs were screened using BLASTn and ABRicate against the PlasmidFinder database (Carattoli et al., 2014). PlasmidSPADES v3.15.5 (Antipov et al., 2016), was also used to attempt plasmid reconstruction; however, we acknowledge its limitations in reliably distinguishing plasmids from chromosomal sequences.

For subtyping purposes Multilocus Sequence Typing (**MLST**) was carried out from the assemblies. Sequence Types (**ST**) and Clonal Complexes (**CC**) were assigned to the *C. coli* strains using the PubMLST scheme v3 (Jolley et al., 2018; https://pubmlst.org/organisms/campylobacter-jejunicoli)

### Statistical analysis

Statistical analyses were performed using R software (R foundation for Statistical Computing, Viena, Austria). The association between the two sampling periods (2010 to 2015 and 2019 to 2020) and resistance frequencies to each antibiotic were calculated. Differences in AR prevalence were assessed using Chi-square or Fisher’s exact test when needed. Likewise, it was also investigated the association between the origin of the strains (extraintestinal vs intestinal) and AR frequencies. Statistical significance was established at p-value < 0,05.

## Results

### Antibiotic susceptibility

Susceptibility to the antibiotics included in the EUCAMP3 plates was tested for the 40 *C. coli* strains (Table 1). All strains were resistant to ciprofloxacin, with 27 out of 40 (67.5%) strains showing high to extremely high levels of resistance (MIC ≥ 16 μg/ml). Resistance to tetracycline (95%) was the second most common, with a high frequency of strains showing moderate-to-high levels of resistance (MIC ≥ 32 μg/ml; 28 out of the 38 strains). Erythromycin resistance was the third most common (53%), and 16 out of the 21 resistant strains were highly resistant (MIC ≥ 512 μg/ml). Lower frequency was observed for ertapenem (28%; MIC ≥ 0,5 μg/ml) and gentamicin (23%; MIC >16 μg/ml). No strains were resistant to chloramphenicol.

**Table 1:** Antibiotic susceptibilities (minimal inhibitory concentration) and resistance mechanisms of *C. coli* from liver and caeca.

Isolates	Source	ST	CC	MIC profile^a^	Quinolone			Tetracycline			Macrolide				
					Cip	R-mech^b^		Tet	R-mech		Ery	R-mech			
						T86I	D90Y		*tet(O)*	*tet(O/32/O)*		23S rDNA	50S	*ermB*	*erm(53)*
1	caeca	854	828	CIP−ERY−TET	R (2)^c^	+	−	R (16)	+	−	R (64)	+	−	−	−
2	caeca	2097	828	CIP−GEN−TET	R (32)	+	−	R (>64)	−	−	S (≤1)	−	−	−	−
3	caeca	854	828	CIP−TET	R (8)	+	−	R (16)	+	−	S (≤1)	−	−	−	−
4	caeca	854	828	CIP−ERY−TET	R (16)	+	−	R (64)	+	−	R (512)	+	−	−	−
5	caeca	827	828	CIP−ETP−TET	R (8)	+	−	R (>64)	+	−	S (≤1)	−	+	−	−
6	caeca	854	828	CIP−ERY−ETP−GEN−TET	R (16)	+	−	R (>64)	+	−	R (>512)	+	−	−	−
7	caeca	854	828	CIP−ERY−TET	R (16)	+	−	R (16)	−	−	R (512)	+	−	−	−
8	caeca	860	828	CIP−ETP−TET	R (32)	+	−	R (>64)	+	−	S (≤1)	−	+	−	−
9	caeca	854	828	CIP−ERY−ETP−TET	R (16)	+	−	R (>64)	+	−	R (>512)	+	−	−	−
10	caeca	14343	828	CIP−ERY−GEN−TET	R (16)	+	−	R (>64)	+	+	R (>512)	−	+	−	−
11	caeca	14697	UN	CIP−ERY−GEN−TET	R (16)	+	−	R (>64)	+	−	R (>512)	+	−	−	−
12	caeca	828	828	CIP−ERY−GEN−TET	R (16)	+	−	R (>64)	+	−	R (>512)	+	−	−	−
13	caeca	854	828	CIP−ERY−TET	R (8)	+	−	R (16)	+	+	R (512)	+	−	−	−
14	caeca	3017	828	CIP−ERY	R (4)	+	−	S (1)	+	−	R (64)	+	−	−	−
15	caeca	854	828	CIP−ERY−TET	R (8)	+	−	R (64)	−	−	R (>512)	+	−	−	−
16	caeca	2177	828	CIP−TET	R (8)	+	−	R (64)	+	−	S (≤1)	−	−	−	−
17	caeca	829	828	CIP−TET	R (16)	+	−	R (64)	+	−	S (≤1)	−	−	−	−
18	caeca	5150	UN	CIP−ERY−ETP−TET	R (>32)	+	−	R (>64)	+	+	R (>512)	+	−	−	−
19	caeca	902	828	CIP−ERY−TET	R (32)	+	−	R (>64)	+	−	R (>512)	+	−	−	−
20	caeca	14699	UN	CIP−TET	R (8)	+	−	R (16)	+	−	S (≤1)	−	−	−	−
21	caeca	14698	UN	CIP−ERY−GEN−TET	R (16)	+	−	R (>64)	−	−	R (>512)	+	+	+	−
22	caeca	854	828	CIP−TET	R (8)	+	−	R (32)	+	−	S (≤1)	−	−	−	−
23	liver	9840	828	CIP−ERY−GEN−TET	R (32)	+	+	R (64)	−	−	R (64)	−	−	−	+
24	liver	902	828	CIP−TET	R (16)	+	−	R (16)	+	−	S (≤1)	−	−	−	−
25	liver	1465	828	CIP−ERY−TET	R (16)	+	−	R (>64)	+	−	R (32)	−	−	−	+
26	liver	1465	828	CIP−ERY−ETP−TET	R (16)	+	−	R (>64)	+	−	R (32)	−	−	−	+
27	liver	6183	828	CIP−ETP−TET	R (8)	+	−	R (>64)	+	−	S (≤1)	−	−	−	−
28	liver	14699	UN	CIP−TET	R (8)	+	−	R (16)	+	−	S (2)	−	−	−	−
29	liver	14699	UN	CIP−TET	R (16)	+	−	R (16)	+	−	S (2)	−	−	−	−
30	liver	1563	828	CIP	R (16)	+	−	S (≤0,5)	−	−	S (2)	−	−	−	−
31	liver	827	828	CIP−ETP−TET	R (16)	+	−	R (>64)	+	−	S (≤1)	−	+	−	−
32	liver	1624	828	CIP−ERY−TET	R (16)	+	−	R (>64)	+	−	R (>512)	−	+	−	−
33	liver	1417	828	CIP−TET	R (8)	+	−	R (32)	+	−	S (≤1)	−	+	−	−
34	liver	14698	UN	CIP−ERY−GEN−TET	R (16)	+	−	R (16)	−	−	R (512)	+	+	+	−
35	liver	3020	828	CIP−ETP−TET	R (8)	+	−	R (>64)	+	−	S (≤1)	−	−	−	−
36	liver	14705	UN	CIP−ERY−TET	R (16)	+	−	R (>64)	+	−	R (>512)	+	−	−	−
37	liver	2097	828	CIP−ERY−GEN−TET	R (16)	+	−	R (>64)	−	−	R (>512)	+	−	−	−
38	liver	854	828	CIP−TET	R (16)	+	−	R (>64)	+	−	S (2)	−	−	−	−
39	liver	832	828	CIP−ETP−TET	R (16)	+	−	R (16)	+	−	S (≤1)	−	+	−	−
40	liver	889	828	CIP−ETP−TET	R (32)	+	−	R (>64)	+	−	S (≤1)	−	−	−	−
				Total (%)	100%			95,00%			53%				

Isolates	Source	ST	CC	MIC profile^a^	β-lactam					Aminoglycoside							
					Etp	R-mech^b^				Gen	R-mech						
						*blaOXA-61*	*blaOXA-184*	*blaOXA-453*	*blaOXA-489*		*aph(2″)*	*aad(6)*	*aadE-Cc*	*ant6-Ia*	*aph(3′)-III*	*aadE*	*sat4*
1	caeca	854	828	CIP−ERY−TET	S (≤0,12) c	−	−	−	−	S (0.5)	−	−	+	−	−	−	−
2	caeca	2097	828	CIP−GEN−TET	S (0,25)	+	−	−	−	R (>16)	−	+	−	+	−	+	−
3	caeca	854	828	CIP−TET	S (≤0,12)	−	−	−	−	S (0.5)	−	−	+	−	−	−	−
4	caeca	854	828	CIP−ERY−TET	S (≤0,12)	−	−	−	−	S (0.5)	−	−	−	−	−	−	−
5	caeca	827	828	CIP−ETP−TET	R (1)	+	−	−	+	S (1)	−	−	+	−	−	−	−
6	caeca	854	828	CIP−ERY−ETP−GEN−TET	R (4)	−	−	−	−	R (>16)	−	−	+	−	+	+	+
7	caeca	854	828	CIP−ERY−TET	S (≤0,12)	−	−	−	−	S (1)	−	−	+	−	−	−	−
8	caeca	860	828	CIP−ETP−TET	R (>4)	+	−	−	+	S (2)	−	−	−	−	−	−	−
9	caeca	854	828	CIP−ERY−ETP−TET	R (0,5)	−	−	−	−	S (1)	−	+	+	+	+	+	+
10	caeca	14343	828	CIP−ERY−GEN−TET	S (0,25)	+	−	−	−	R (>16)	+	−	+	−	−	−	−
11	caeca	14697	UN	CIP−ERY−GEN−TET	S (≤0,12)	−	−	−	−	R (>16)	+	−	+	−	+	−	+
12	caeca	828	828	CIP−ERY−GEN−TET	S (≤0,12)	−	−	−	−	R (>16)	+	−	+	+	+	−	+
13	caeca	854	828	CIP−ERY−TET	S (≤0,12)	−	−	−	−	S (≤0,25)	−	−	+	−	−	−	−
14	caeca	3017	828	CIP−ERY	S (≤0,12)	+	−	−	−	S (≤0,25)	−	−	−	−	−	−	−
15	caeca	854	828	CIP−ERY−TET	S (≤0,12)	−	−	−	−	S (0.5)	−	+	+	+	+	+	+
16	caeca	2177	828	CIP−TET	S (≤0,12)	+	−	−	−	S (0.5)	−	+	−	+	−	+	−
17	caeca	829	828	CIP−TET	S (≤0,12)	−	+	−	−	S (0.5)	−	+	+	+	−	+	−
18	caeca	5150	UN	CIP−ERY−ETP−TET	R (0.5)	+	−	−	+	S (1)	−	−	−	−	−	−	−
19	caeca	902	828	CIP−ERY−TET	S (≤0,12)	−	−	−	−	S (1)	−	−	−	−	−	−	−
20	caeca	14699	UN	CIP−TET	S (≤0,12)	−	−	−	−	S (0.5)	−	−	+	−	−	−	−
21	caeca	14698	UN	CIP−ERY−GEN−TET	S (0.25)	+	−	−	+	R (>16)	+	+	−	+	+	+	−
22	caeca	854	828	CIP−TET	S (≤0,12)	+	−	−	−	S (0.5)	−	+	+	+	+	+	+
23	liver	9840	828	CIP−ERY−GEN−TET	S (≤0,12)	+	−	−	−	R (>16)	+	+	−	+	+	+	−
24	liver	902	828	CIP−TET	S (≤0,12)	−	−	−	−	S (0.5)	−	−	−	−	−	−	−
25	liver	1465	828	CIP−ERY−TET	S (0,25)	+	−	−	−	S (0.5)	−	+	−	+	+	+	−
26	liver	1465	828	CIP−ERY−ETP−TET	R (0,5)	+	−	−	−	S (1)	−	+	−	+	+	+	−
27	liver	6183	828	CIP−ETP−TET	R (2)	+	−	−	+	S (1)	−	−	−	−	−	−	−
28	liver	14699	UN	CIP−TET	S (≤0,12)	−	−	−	−	S (0.5)	−	−	+	−	−	−	−
29	liver	14699	UN	CIP−TET	S (≤0,12)	−	−	−	−	S (0.5)	−	−	+	−	−	−	−
30	liver	1563	828	CIP	S (≤0,12)	+	−	−	−	S (0.5)	−	−	−	−	−	−	−
31	liver	827	828	CIP−ETP−TET	R (2)	+	−	−	−	S (1)	−	−	+	−	−	−	−
32	liver	1624	828	CIP−ERY−TET	S (≤0,12)	+	−	−	−	S (0.5)	−	−	+	+	+	+	−
33	liver	1417	828	CIP−TET	S (0,25)	−	−	−	−	S (1)	−	−	+	−	−	−	−
34	liver	14698	UN	CIP−ERY−GEN−TET	S (≤0,12)	+	−	−	+	R (>16)	+	+	−	+	+	+	−
35	liver	3020	828	CIP−ETP−TET	R (1)	−	−	−	−	S (0,5)	−	+	−	+	+	−	+
36	liver	14705	UN	CIP−ERY−TET	S (0.25)	+	−	−	−	S (2)	−	−	−	−	−	−	−
37	liver	2097	828	CIP−ERY−GEN−TET	S (≤0,12)	+	−	−	−	R (>16)	+	+	−	+	+	+	−
38	liver	854	828	CIP−TET	S (0.25)	+	−	−	−	S (2)	−	+	+	+	+	+	+
39	liver	832	828	CIP−ETP−TET	R (0.5)	+	−	−	−	S (0.5)	−	−	+	−	−	−	−
40	liver	889	828	CIP−ETP−TET	R (1)	+	−	+	+	S (1)	−	−	+	−	−	+	−
				Total (%)	28%					23%							

a MIC profile: CIP, ciprofloxacin; ETP, ertapenem; ERY erythromycin; GEN, gentamicin; TET, tetracycline.

b R-mech: resistance mechanisms. T86I, D90Y: point mutations in the subunit A of the gyrase gene. *Tet(O), Tet(O/32/O), ermB, erm(53), blaOXA-*61 (*blaOXA-*61 family which includes: *blaOXA-61, −193, −450, −460, −461, −605*), blaOXA*-184, blaOXA-453, blaOXA-489, aad(6), aadE, aad-Cc, ant6-Ia, aph(2″), aph(3′)-III, sat4*: presence of these genes. 23S rRNA: point mutation A2075G; 50S: A103V point mutation in the ribosomal protein L22 gene.

c Interpretation of the MIC values for *C. coli* epidemiological cut-off values: CIP (R > 0,5mg/L), ERY (R > 8mg/L), ETP (R > 8mg/L), GEN (R > 2mg/L), TET (R > 2mg/L).

**Fig. 1:**
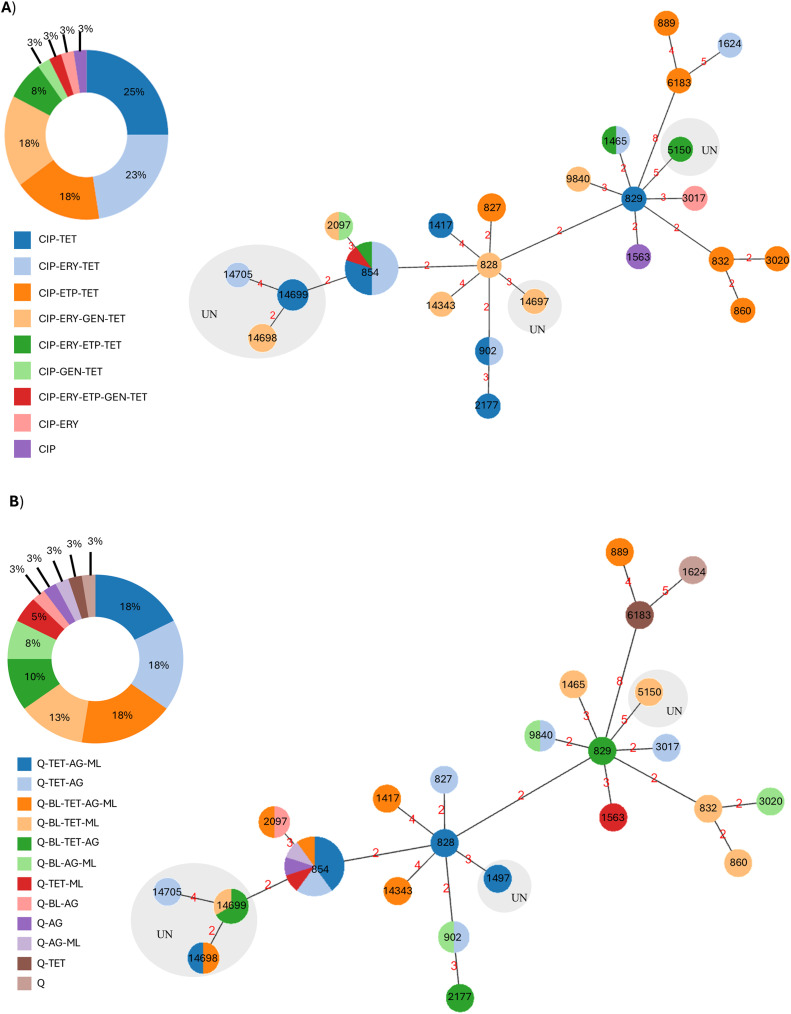
Minimum spanning tree of *C. coli* isolates constructed from MLST profiles showing the distribution of: (**A**) phenotypic resistance profiles obtained from the MIC analysis and (**B**) predicted resistance to antibiotic families based on WGS analysis. Each node represents a sequence type (ST), and its size correlates with the number of isolates belonging to the ST. Branches link STs with one or more different loci, and the number of differences is indicated on the branches. Unassigned (UN) clonal complexes (CC) with more than one ST are highlighted with a grey shadow; the remaining STs belong to the CC-828. Antibiotics: CIP, ciprofloxacin; ETP, ertapenem; ERY, erythromycin; GEN, gentamicin; TET, tetracycline. Antimicrobial families: AG, aminoglycosides; BL, β-lactams; ML, macrolides; Q, quinolones. Antimicrobial resistance profiles shown as percentages are given in separate pie charts. This figure was created using https://www.phyloviz.net/.

The AMR profile CIP-TET was the most frequent (25%), followed by CIP-TET-ERY (23%). Overall, 70% of the strains were MDR, with resistances up to 5 classes of antibiotics (Fig. 1A).

Statistical analysis unveiled no differences between the AR frequencies when stratified by sampling periods (between 2010 and 2015 and between 2019 and 2020; p > 0.05). Similarly, no differences in the occurrence of AR were observed according to strain origin (intestinal vs. extraintestinal; p > 0.05).

### WGS Analysis

All 40 strains were WGS and assembled and an average of 24.07 contigs per isolate was obtained with a mean assembly size of 1,737,055 (Supplementary Table 1).

To explore the potential mechanisms of AR, the assembled genomes of each of the *C. coli* strains were investigated for genes and/or mutations known to be associated to resistance. All isolates showing phenotypic ciprofloxacin resistance carried a point mutation in the subunit A of the DNA gyrase gene (C257T) resulting in a threonine (**T**) to isoleucine (**I**) substitution in the gyrase, commonly referred to as p.T86I (Table 1). This mutation is well known to confer resistance to quinolones. One strain showed a second point mutation in the *gyrA* gene (p.D90Y), also associated with quinolone resistance. Additionally, multiple point non-synonymous mutations in the *gyrA* gene (p.A206T, p.A664V, p.F723L, p.L483P, p.N404S, p.R285K, p.S22G, p.T269I, p.T665S, p.V149I) with an unknown role were detected in several strains; (Supplementary Table 2).

Regarding the 38 tetracycline-resistant strains, all but seven carried the chromosomally encoded *tet(O)* gene, and three strains carried additionally the *tet(O/32/O)* gene (Table 1). The chromosomally encoded *tet(O)* gene was also identified in one phenotypically susceptible strain. Since tetracycline resistance can also be plasmid-encoded, we investigated the presence of plasmid-encoded tetracycline-resistance genes using ABRicate, BLASTn, and PlasmidFinder, but none were identified.

The mutation A2075G in the 23S rRNA gene conferring resistance to different macrolides, including erythromycin, was present in 16 out of the 21 erythromycin-resistant strains. The phenotypic resistance observed in the five remaining strains can be explained by the presence of the point mutation p.A103V in the 50S protein L22 or the presence of the *erm(53)* gene. Two resistant strains carried the *erm(B)* gene and the above-mentioned point mutations p.A2075G and p.A103V in the 23S rDNA and 50Sprotein L22, respectively. Besides, the p.A103V mutation in the 50S protein L22 was also identified in five erythromycin-susceptible strains. Additionally, multiple point mutations in the 23S rRNA gene with an unknown role were detected in 20 strains (Supplementary Table 2).

Phenotypic resistance to ertapenem was observed in 11 strains (28%), but no specific carbapenem resistance mechanism was identified; however, these resistant strains carried *blaOXA* genes associated with β-lactam resistance (*blaOXA-61, blaOXA-453* or *blaOXA-489*). Also, a G57T mutation in the promoter region of the *blaOXA*-489 gene was identified in four strains (8, 18, 21 and 34). Overall, among the 40 studied strains, 68% carried at least one of the four *blaOXA* genes identified, with *blaOXA-61* being the most prevalent (55% of the strains) (Table 1).

Concerning aminoglycosides, seven of the nine gentamicin-resistant strains carried the *aph(2″)* gene, known to confer resistance to gentamicin. Additionally, other genes associated with resistance to aminoglycosides were detected in both the gentamicin-resistant and 22 of 31 susceptible strains (Table 1).

All strains were susceptible to chloramphenicol; accordingly, no phenicol resistance mechanism was detected in any of the strains. On the contrary, the genes coding for the efflux pumps cmeABC and cmeDEF were present in all strains. The phenotypic resistance seen in most strains corresponded with the WGS predicted profiles, showing a strong consistency between the phenotypes and genotypes.

**Fig. 2:**
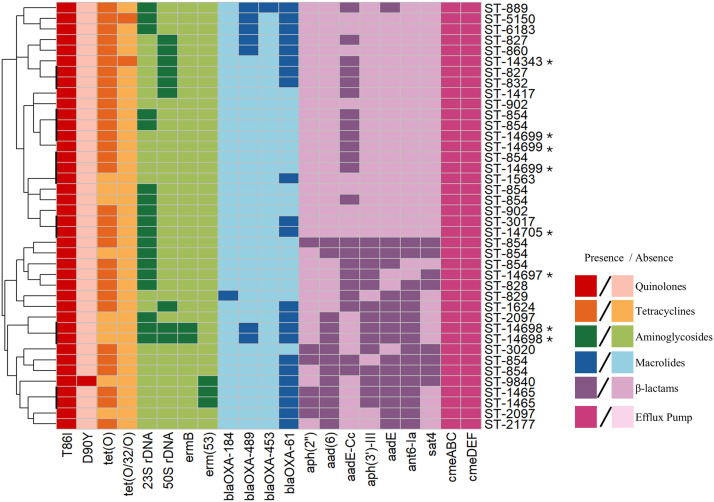
Heatmap of presence/absence of resistance determinants for each strain. On the right, their sequence type (ST). *Indicative of new ST.

The genome-inferred MLST analysis revealed 24 different sequence types (ST; Table 3, Fig. 2), with four of them being novel (ST14697, ST14698, ST14699 and ST14705) not previously reported in the PubMLST database (https://pubmlst.org/organisms/campylobacter-jejunicoli). Of these, two novel STs arose from new combinations of existing alleles (ST14697 and ST14698), while the other two resulted from the identification of previously unreported alleles (ST14699, new allele *uncA* and ST14705, new allele *aspA)*. ST854 was the most frequent (25%) and most STs belonged to the CC 828, while only five STs were associated to unassigned CCs.

In addition to sequence typing, WGS identified specific resistance determinants to antibiotics not included in the EUCAMP3 plates. Hence, overall, WGS analysis predicted 92.5% (37 out of 40) of the strains as MDR (Table 2; Fig. 1B). These included resistance determinants to several aminoglycosides such as amikacin, butirosin, hygromycin, kanamycin, lividomycin, neomycin, paromomycin, ribostamycin, spectinomycin, streptomycin, streptothricin and tobramycin. The *aadE-Cc* gene conferring resistance to aminoglycosides was the most common (55%) and was mainly present in ST-854, ST-1465 and ST-14699. The *aadE- sat4- aph(3′)-III* cassette was present in 12.5% of strains, all of which belonged to ST-854 (Fig. 2). Only nine strains lacked genes associated with aminoglycoside resistance, and belonged to ST-854, ST-860, ST-902, ST-1563, ST-3017, ST-5150, ST-6183, and the novel ST-14705 (Fig. 2). The most common predicted pattern of AR revealed by WGS included resistance mechanisms to quinolones including nalidixic acid and ciprofloxacin; tetracyclines like doxycycline, minocycline, and tetracycline; β-lactam antibiotics such as amoxicillin and ampicillin and macrolides like erythromycin. This AR profile (CipNalAmoAmpDoxyMinoTetEry; Table 2) was observed in five isolates belonging to different STs: ST-832, ST-860, ST-3017, ST-5150 and ST-6183. The only strain presenting the largest phenotypic resistance profile (5 antibiotics) corresponds to the ST-854, and the single strain presenting resistance only to ciprofloxacin belonged to the ST-1563 (Fig. 1B). The most prevalent STs (ST-854) with 10 strains was present across 9 different farms; the second most frequent STs was ST-14699 (3 strains) and was present in 2 different farms. According to the pubMLST database, the most frequent sources of the STs from the intestinal and extraintestinal *C. coli* strains were human stools followed by chicken and chicken products (Table 3). However, few STs – most notably the prevalent ST-854, along with ST-1417, ST-1465, ST-1563, and ST-1624 – have swine as a highly frequent source.

**Table 2:** Antimicrobial resistance profiles predicted by WGS related to their sequence type (ST) and clonal complex (CC). New ST are shown in bold.

AMR Predicted pattern^a^	N atb^b^	N^c^	CC (ST)^d^
CipNalDoxyMinoTetAmiButGenHigKanLivNeoParRibSptxStnStrStnEry	4	1	UN (**14697**)
CipNalAmoAmpDoxyMinoTetButGenKanLivNeoParRibSptStrTobEry	5	1	828 (2097)
CipNalDoxyMinoTetAmiButGenKanLivNeoParRibSptStrStnTobEry	4	1	828 (854)
CipNalDoxyMinoTetAmiButGenHigKanLivNeoParRibSptStnStrEry	4	1	828 (828)
CipNalAmoAmpAmiButGenKanLivNeoParRibSptStrTobEry	4	2	UN (**14698**)
CipNalAmoAmpDoxyMinoTetButKanLivNeoParRibStrEry	5	1	828 (1624)
CipNalAmoAmpAmiButGenKanLivNeoParRibTobSptStr	3	1	828 (2097)
CipNalAmoAmpDoxyMinoTetButKanLivNeoParRibStr	4	1	828 (1465)
CipNalAmoAmpDoxyMinoTetButKanLivNeoParStrStr	4	1	828 (1465)
CipNalAmoAmpAmiButHigKanLivNeoLivRibStpStrEry	4	1	828 (9840)
CipNalAmoAmpDoxyMinoTetAmiGenKanStrStnEry	5	1	828 (**14343**)
CipNalDoxyMinoTetButKanLivNeoParRibSptStnStr	3	1	828 (854)
CipNalDoxyMinoTetButKanLivNeoRibParSptStnStr	3	1	828 (854)
CipNalDoxyMinoTetButKanLivNeoParRibStnStr	3	1	828 (3020)
CipNalDoxyMinoTetButLivNeoParRibStnStrEry	4	1	828 (854)
CipNalButHigKanLivNeoParRibSptStnStrEry	3	1	828 (854)
CipNalAmoAmpDoxyMinoTetSptStrEry	5	1	828 (827)
CipNalAmoAmpDoxyMinoTetSptStr	4	2	828 (829, 2177)
CipNalAmoAmpDoxyMinoTetStnEry	5	1	UN (**14705**)
CipNalAmoAmpDoxyMinoTetStrEry	5	2	828 (827,889)
CipNalAmoAmpDoxyMinoTetEry	4	5	828 (832, 860, 3017, 6187), UN (5150)
CipNalDoxyMinoTetStrEry	4	3	828 (854, 1417)
CipNalDoxyMinoTetEry	3	2	828 (854, 902)
CipNalDoxyMinoTetStr	3	4	828 (854), UN (**14699**)
CipNalSptStnStrEry	2	1	828 (854)
CipNalDoxyMinoTet	2	1	828 (902)
CipNalAmoAmp	2	1	828 (1563)

a Antimicrobial resistance (AMR) pattern: Aminoglycosides (Ami, amikacin; But, butirosin; Gen, gentamicin; Hyg, hygromycin; Kan, kanamycin; Liv, lividomycin; Neo, neomycin; Par, paromomycin; Rib, ribostamycin; Spt, spectinomycin; Stn, streptothricin; Str, streptomycin; Tob, tobramycin); β-lactams (Amo, amoxicillin; Amp, ampicillin); Macrolide (Ery, erythromycin); Quinolones (Cip, ciprofloxacin; Nal, nalidixic acid); Tetracyclines (Doxy, doxycycline; Mino, minocycline; Tet, tetracycline).

b N atb: Number of classes of antimicrobial agents; resistance to three or more classes of antimicrobial agents, indicative of multidrug resistance.

c N: Number of *Campylobacter* isolates per AMR pattern.

d UN: unassigned CC.

**Table 3:** Sequence types (ST) of *C. coli* isolates from this study compared with the wider population of STs of the PubMLST database. The most frequent sources are shown in bold. Data updated in September 2025.

ST (n)^a^	PubMLST isolates^b^	Human Stool	Chicken	Chicken offal or meat	Pig	Cattle	Other animal	Human unspecified
827(2)	1917	**760 (40%)**	344 (17,9%)	101 (5,3%)	20 (1%)	120 (2,3%)	18 (0,9%)	47 (2,5%)
828(1)	1038	90 (8,7%)	**433 (41,7%)**	206 (19,8%)	171 (16,5%)	40 (3,9%)	2 (0,2%)	21 (2%)
829 (2)	2832	155 (5,5%)	**1039 (36,7%)**	**986 (34,8%)**	69 (2,4%)	31 (1,1%)	4 (0,1%)	89 (3,1%)
832(1)	142	**44 (31%)**	15 (10,6%)	**36 (25,4%)**	3 (2%)	2 (1,4%)	−	18 (12,7%)
854(10)	670	40 (6%)	**188 (28,1%)**	46 (6,9%)	**283 (42,2%)**	17 (2,5%)	6 (0,9%)	12 (1,8%)
860 (1)	176	**72 (40,9%)**	**54 (30,7%)**	14 (8%)	−	−	4 (2,3%)	18 (10,2%)
889 (1)	50	2 (4%)	**19 (38%)**	**13 (26%)**	−	1 (2%)	−	1 (2%)
902(2)	409	9 (2,2%)	49 (12%)	25 (6,1%)	8 (2%)	**224 (55%)**	−	40 (9,8%)
1417(1)	18	2 (11,1%)	−	1 (5,6%)	**7 (38,9%)**	−	**7 (38,9%)**	−
1465(2)	7	−	−	1 (14,3%)	**5 (71,4%)**	−	1 (14,3%)	−
1563 (1)	15	2 (13,3%)	1 (6,7%)	**42 (67%)**	**6 (40%)**	1 (6,7%)	−	−
1624 (1)	25	1 (4%)	−	1 (4%)	**17 (68%)**	−	−	1 (4%)
2097 (2)	8	**3 (37,5%)**	**3 (37,5%)**	−	8 (2%)	−	−	−
2177 (1)	10	2 (20%)	−	**4 (40%)**	−	−	−	2 (20%)
3017(1)	17	**9 (53%)**	−	1 (6%)	−	3 (18%)	−	1 (6%)
3020 (1)	8	**3 (38%)**	**4 (50%)**	−	−	−	−	1 (12,5%)
5150 (1)	33	**21 (63,6%)**	2 (6,1%)	1 (3%)	−	−	1 (3%)	5 (15,2%)
6183 (1)	4	**1 (25%)**	−	**2 (50%)**	−	−	−	−
14343(1)	1	−	**1 (100%)**	−	−	−	−	−
9840 (1)	1	−	−	−	−	−	−	−

a n: in parentheses total number of isolates from this study belonging to each ST; ST-9840, source not identified in the pubMLST database.

b PubMLST isolates: total number of isolates in the pubMLST database.

## Discussion

The high prevalence of AR *Campylobacter* strains in food-producing animals has been associated with an increased incidence of corresponding resistant infections in humans (ECDC, EFSA and EMA, 2024). Therefore, surveillance of AR *Campylobacter* in food-producing animals, particularly poultry and poultry products, is essential for public health. In Spain, a high frequency of AR *Campylobacter* has been reported (Bort et al., 2022; Marin et al., 2020), as in other EU countries (Barata et al., 2024; EFSA and ECDC, 2025b). While AR in *Campylobacter* from poultry and poultry meat has been widely studied, information on *Campylobacter* AR from avian edible tissues, such as livers, remains limited (Noormohamed and Fakhr, 2012; Simaluiza et al., 2015), despite this organ has been shown to be a relevant source of *Campylobacter* (Manzanares-Pedrosa et al., 2025b; Prendergast et al., 2025), with only a few studies focused on *C. jejuni*, leaving a dearth of knowledge on *C. coli* (Karki et al., 2019). The present study expands current knowledge on the occurrence of AR *C. coli* strains in broiler chickens, including its detection in internal tissue liver and supports the use of WGS data as a reliable predictor of AR.

The World Health Organization (WHO) classifies fluoroquinolones and macrolides as critically important antibiotics for human medicine (WHO, 2019), as they are the primary treatment options for severe *Campylobacter* infections in humans (Dai et al., 2020). Tetracyclines are considered alternative therapeutic options for *Campylobacter* infections and are categorized by the WHO as highly important antibiotics, while aminoglycosides are recommended for treating *Campylobacter* bacteremia (Moore et al., 2006). Consequently, a high prevalence of AR *Campylobacter*, especially to antibiotics classified as critically important by WHO would constitute a significant public health concern.

Here, ciprofloxacin resistance was the most prevalent among *C. coli* isolates, since all isolates showed phenotypic resistance, and carried the point mutation p.T86I in the *gyrA* gene. This mutation has been reported to confer high levels of resistance to quinolones (Engberg et al., 2001; Luo et al., 2005), and consequently, a high proportion of isolates showed a high to extremely high levels of resistance. One isolate additionally harbored the point mutation p.D90Y in the *gyrA* gene, typically conferring lower level of resistance to quinolones (Engberg et al., 2001). The less common p.A70T, p.D90N and p.T86K mutations were not detected in the strains examined in this study (Wieczorek and Osek, 2013). However, we identified up to ten previously unreported point mutations within the same region of the *gyrA* gene. The most common was p.A206T, present in five isolates and resulting from a GCT to ACT nucleotide substitution, which leads to the replacement of alanine with threonine (Supplementary Table 2). This point mutation, together with the p.S22G, p.R285K and p.T665S substitutions of unknown role in fluoroquinolone resistance, was previously detected at high frequency in *C. jejuni* isolates from chicken livers obtained in the same sampling (Manzanares-Pedrosa, et al., 2025a). Further research is needed to confirm the role of these point mutations in ciprofloxacin resistance. The extremely high prevalence of fluoroquinolone-resistant *C. coli* isolates we have found is consistent with previous findings in poultry and poultry meat from Spain (Cantero et al., 2018; Nafarrate et al., 2021) and what is reported across EU countries (EFSA and ECDC, 2025b). Moreover, ciprofloxacin resistance has been reported to enhance the strain fitness even in the absence of the antibiotic selective pressure (Luo et al., 2005), suggesting that the rapid emergence of fluoroquinolone-resistant *Campylobacter* on a worldwide scale may be attributable partly to the enhanced fitness of the fluoroquinolone-resistant isolates.

Resistance to tetracycline was the second most prevalent phenotype (95%) and was mostly explained by the presence of the chromosomal *tet(O)* gene and *tet(O/32/O)* mosaic gene. The frequencies of these resistance determinants were similar to those we observed in the *C. jejuni* strains isolated from the same samples (Manzanares-Pedrosa et al., 2025a). This contrasts with the findings of Prendergast et al. (2025), who reported lower frequencies of *tet(O)* but higher frequencies of the mosaic gene, which might be indicative of geographic variation of these resistance determinants. The *tet(O/32/O)* was first described in *C. coli* and *C. jejuni* isolated from humans and is a functional chimera containing a sequence of about 300 bp from the *tet(32)* gene inserted in the *tet(O)* gene (Warburton et al., 2016). Its prevalence is rapidly increasing with up to 70% of clinical tetracycline-resistant isolates in EU in 2021 (EFSA and ECDC, 2023). While plasmids like pTet, encoding the *tet(O)* gene have been described in *Campylobacter* (García-Fernández et al., 2024; He et al., 2025), no plasmids carrying tetracycline resistance genes could be identified in the present study, since the extraction method used is more focused on the genomic DNA and is not optimized for plasmids isolation. Tetracycline resistance in those strains lacking the *tet(O)* gene might be explained by other mechanisms, such as the cmeABC multidrug efflux pump and to a lower extent to cmeDEF (Shen et al., 2018). The cmeABC efflux pump is the best described multidrug efflux pump to date and it is a major mechanism utilized by *Campylobacter* for both intrinsic and acquired antibiotic resistance (Marotta et al., 2019). It has evolved in *Campylobacter* in response to antibiotic selection pressure, contributing to resistance to different antibiotics such as tetracycline, but also erythromycin and ciprofloxacin (Dai et al., 2024), whereas cmeDEF appears to have a minor involvement in AR resistance (Shen et al., 2018). The high prevalence of tetracycline resistance we have found is in line with previous findings in poultry and poultry products in Spain (Melero et al., 2012; Pérez-Boto et al., 2014; Bort et al., 2022) as well as in other EU countries, such as Poland (Wieczorek et al., 2018) and worldwide (Varga et al., 2019; Yao et al., 2020).

Fifty-three percent of the isolates were resistant to erythromycin, with a high proportion showing high resistance levels (MIC >512 mg/L). Consistently, the point mutation A2075G in the 23S rDNA region that has been reported as the most common conferring high erythromycin-resistance levels (Wieczorek and Osek, 2013), was present in all but 5 resistant strains. Besides, a point mutation in the protein L22 of the 50S subunit was present in 23% of the strains, although 5 (55%) of them were susceptible to erythromycin. However, resistance conferred alone by variations in the 50S subunit has been demonstrated to be minimal, and the effect of the cmeABC efflux pump was needed together with this mutation to observe macrolide-resistant phenotypes (De Fátima Rauber Würfel et al., 2020). None of the aforementioned resistance determinants were detected in three isolates exhibiting phenotypic resistance to erythromycin. Instead, resistance was attributable to the presence of the gene *erm(53)* (also referred as *erm(N)*), while *erm(B)* was detected in two resistant strains. *Erm(B)* has been reported as sufficient to confer very high levels of macrolide resistance (Qin et al., 2014; Jehanne et al., 2021); it has been described both in *C. coli* and *C. jejuni* (Jehanne et al., 2021), but in Europe it has only been reported in *C. coli* isolates from broilers and turkeys (Florez-Cuadrado et al., 2016; Elhadidy et al., 2019). The recently described *erm(N)* gene has only been described in Canada, France and Italy in food-animal and clinical isolates (García-Fernández et al., 2025; Jehanne et al., 2025), and it is associated with heterogeneous levels of resistance to erythromycin (MICs ranging from 16 to 512 mg/L). To our knowledge, this study reports for the first time the identification of *erm(53)*/*erm(N)* in *C. coli* strains from poultry in Spain, mediating moderate erythromycin resistance levels (32 to 64 µg/ml). Macrolide resistance is more frequent in *C. coli* strains than in *C. jejuni* both in humans and animals (EFSA and ECDC, 2025b)*.* The high prevalence of macrolide resistance is of particular concern, as macrolides are classified as critically important antibiotics (**CIA**) for human medicine and represent the treatment of choice for *Campylobacter* infections when antimicrobial therapy is required. The increasing resistance and the spread of resistance mechanisms such as *erm(B)* or *erm(N)* reported in recent years threatens the effectiveness of these treatments (Dai et al., 2020; ECDC, EFSA and EMA, 2024). Combined resistance to CIP and ERY was highly prevalent (52.5%), in contrast to the low levels reported in broilers by EFSA and ECDC (2025b). This finding is of concern to public health, since simultaneous resistance to both drugs might hamper the main therapeutic options for severe human campylobacteriosis.

Ertapenem (carbapenem) was the only representative of β-lactam antibiotics in the EUCAMP3 plates. Resistance was found in 28% of the isolates; among them, all but three isolates carried at least one *blaOXA* gene. The most frequent were *blaOXA-61* and *blaOXA-489* (55% and 18% respectively), typically providing β-lactam resistance in *C. coli* and also being reported as the most frequent in this species (Cobo-Díaz et al., 2021; Yang et al., 2025) although with an uncertain role in carbapenem resistance (Griggs et al., 2009; Zhuo et al., 2024). However, it has been suggested that the efflux pump cmeABC together with an overexpression of *blaOXA*-61 may lead to resistance to ertapenem (Maurille et al., 2024). On the other hand, the G57T mutation in the promoter of the *blaOXA*-489 gene has been suggested to contribute to carbapenem resistance (Maurille et al., 2024), however, it was present in both ertapenem-resistant and susceptible strains. Hence, this mutation might not be sufficient on its own to produce the resistant phenotype. Ertapenem resistance in *Campylobacter* has been associated with mutations of the major outer membrane protein *porA* gene (Bonilla-Moreno et al., 2023; Nunes et al., 2023) or the presence of genes like *blaVIM, blaNDM-1, blaIMP, blaOXA-48* (Okello et al., 2023) or presumably *blaOXA-185* (Manzanares-Pedrosa et al., 2025a). However, we did not find such mechanisms in our set of strains. For the remaining three isolates not harboring blaOXA genes, resistance might be mediated by the CmeABC efflux pump. Overall, 68% of the isolates carried at least one of the following β-lactam-resistance genes: *blaOXA-61, blaOXA-184, blaOXA-453, blaOXA-489*, with all but the latter also identified in *C. jejuni* isolates from the same sampling (Manzanares-Pedrosa et al., 2025a). *BlaOXA-61* is widely distributed worldwide both in *C. jejuni* and *C. coli* (Cobo-Díaz et al., 2021), and accordingly it was the most prevalent in this study. *BlaOXA-489* is prevalent in ruminants in northern Spain (Ocejo et al., 2021), suggesting a regional circulation of this β-lactamase variant across different livestock reservoirs. Nevertheless, the lack of representation of other β-lactam antibiotics in EUCAMP3 plates apart from carbapenems, leaves a gap in the correlation between phenotype and genotype. Carbapenems are on the list of CIA (WHO, 2024). Therefore, *C. coli* of animal origin that are phenotypically resistant to carbapenems or carry genetic determinants potentially conferring resistance may compromise the use of these antimicrobials for the treatment of human infections. This is particularly concerning given the high levels of resistance we have found and also reported by EFSA and ECDC (2025b).

Aminoglycosides were represented phenotypically by gentamicin to which 23% of the strains were resistant. While the *aph(2″)* responsible for gentamicin resistance was present in all but three resistant isolates, it is unclear if any of the additional genes identified associated with aminoglycoside resistance or the efflux pumps contributed to the resistance of those isolates. *AadE-Cc* was the most frequently identified gene, occurring in 22 isolates. This gene has a major role in aminoglycoside resistance in *C. coli* (Zhang et al., 2022) and has been found with high frequency in *C. coli* strains from ruminants in Spain (Ocejo et al., 2021). The cassette *aadE- sat4- aph(3′)-III*, present in 12.5% of the isolates, is recognized to confer resistance to streptomycin, streptothricin, and kanamycin and can be plasmid or chromosomally encoded (Zhang et al., 2021). The increasing prevalence of this cassette in *C. coli*, together with its ability to be horizontally transferred to *C. jejuni* may facilitate the dissemination of aminoglycoside resistance between *Campylobacter* species, potentially contributing to the emergence of MDR strains (Qin et al., 2012). Both phenotypic gentamicin resistance and the resistance genes identified here have been reported to occur more frequently in *C. coli* than in *C. jejuni* (Bai et al., 2024). In agreement with these findings, we observed the same pattern when comparing *C. coli* (this study) and *C. jejuni* strains obtained from the same sampling (Manzanares-Pedrosa et al., 2025a). The use of aminoglycoside antibiotics in the poultry industry might play an important role in the dissemination and positive selection of these resistance genes (Zhang et al., 2021). In the EU, gentamicin resistance in *C. coli* from both humans and food-producing animals is regarded as moderate to high (EFSA and ECDC, 2025b). Aminoglycosides are used in the treatment of sporadic systemic *Campylobacter* infections in humans; therefore, the presence and dissemination of the aforementioned genes, or other determinants conferring aminoglycoside resistance, may pose a risk to public health.

Differences were observed in the detection of MDR strains. Phenotypically, 70% of the strains were classified as MDR (Fig. 1A), whereas WGS predicted 92.5% to be MDR (Table 2, Fig. 1B). The higher AR prevalence predicted by WGS is attributable to the absence of certain antibiotics, such as amoxicillin, ampicillin, and streptomycin, on the EUCAMP3 plates, which are nevertheless detected at the genomic level, adding value to the usefulness of WGS data as a predictor for AR. The present findings of MDR *C. coli,* support the fact that MDR strains are more commonly found in *C. coli* than *C. jejuni* (EFSA and ECDC, 2023; Manzanares-Pedrosa et al., 2025a).

Moreover, WGS provided information on the *C. coli* genotypes. According to the PubMLST, STs from this study have been associated with human stool and poultry sources (including chicken offal and meat) across multiple countries, highlighting the public health relevance of *Campylobacter*, and emphasizing that the risk is associated not only from poultry meat but also with offal.

In contrast to *C. jejuni*, which exhibits greater ST and CC diversity, *C. coli* isolates tend to be genetically more conserved and most of them belong to the host generalist CC828 (Prendergast et al., 2025); accordingly, nearly all isolates in this study were assigned to this CC, which has also been associated to clinical isolates (https://pubmlst.org/organisms/campylobacter-jejunicoli). The ST-827 and ST-2097 identified in this study have previously been reported in ruminants from northern Spain (Ocejo et al., 2021), suggesting a geographical spread of these lineages and that they are not restricted to a single animal reservoir.

Analysis of the association between STs and AMR profiles predicted by WGS revealed no consistent link between specific STs and particular resistance patterns (Table 2; Figs. 1B and 2), in agreement to previous studies (Zarske et al., 2024; Manzanares-Pedrosa et al., 2025b).

Since continuous monitoring of AR and investigation of their underlying mechanisms are crucial to combat its spread (Marotta et al., 2019), this study provides insights into the development of AR in *C. coli* strains in two different time points. Although no differences were observed over a ten-year period, an eventual decrease in the occurrence of AR is expected as a positive outcome of efforts to reduce the antibiotics use such as the National Plan for Antibiotic Resistance in Spain, active since 2014, which has led to a 70% reduction in antibiotic consumption in the poultry sector (PRAN, 2019). However, despite reduced antibiotic use, resistance determinants to some antibiotics such as quinolones or tetracyclines (the most frequent in our isolates) may still persist when they do not incur a fitness cost (Luo et al., 2005).

While this study provides valuable insights, it has some limitations. The relatively limited size of the present study (40 isolates) and the lack of relevant antibiotics in the EUCAMP3 plates such as β-lactams, limit comprehensive genotype–phenotype correlations and restricts the scope of the landscape of AR in *C. coli.* Also, further functional studies are required to validate the role of novel mutations and to confirm both the expression and the phenotypic impact of the identified resistance determinants.

Overall, this study underscores the public health risk posed by both chickens and chicken livers as reservoirs of AR *C. coli* and highlights WGS as a rapid, reliable tool for monitoring and predicting resistance, which is essential for effective surveillance and control strategies.

## Data availability statement

The raw sequence data set presented in this study is available in the NCBI database online repositories. The name of the BioProject accession number(s) can be found below: PRJNA1440180.

## CRediT authorship contribution statement

**Mª Pilar González-Navarro:** Writing – original draft, Investigation. **Alicia Manzanares-Pedrosa:** Investigation, Formal analysis, Data curation. **Florencia Correa-Fiz:** Writing – review & editing, Validation, Supervision, Methodology. **Lauge Holm Sørensen:** Writing – review & editing, Supervision. **Teresa Ayats:** Methodology. **Núria Aloy:** Methodology. **Miquel Nofrarías:** Writing – review & editing, Supervision, Project administration. **Rene S. Hendriksen:** Writing – review & editing, Supervision. **Marta Cerdà-Cuéllar:** Writing – review & editing, Supervision, Resources, Project administration.

## Disclosures

The authors declare that they have no known competing financial interests or personal relationships that could have appeared to influence the work reported in this paper.

## Appendix Supplementary materials

Image, application 1

## Footnotes

Supplementary material associated with this article can be found, in the online version, at doi:10.1016/j.psj.2026.107271.

## References

[bib0001] Akiba M., Lin J., Barton Y.-W., Zhang Q. (2006). Interaction of CmeABC and CmeDEF in conferring antimicrobial resistance and maintaining cell viability in *Campylobacter jejuni*. J. Antimicrob. Chemother..

[bib0002] Antipov D., Hartwick N., Shen M., Raiko M., Lapidus A., Pevzner P.A. (2016). plasmidSPAdes: assembling plasmids from whole genome sequencing data. Bioinformatics.

[bib0003] Bai Y., Ma J., Li F., Yang B., Ren X., Wang Y., Hu Y., Dong Y., Wang W., Zhang J., Yan S., Cui S. (2024). Antimicrobial resistance and genomic characterization of *Campylobacter jejuni* and *Campylobacter coli* isolated from retail chickens in Beijing, China. Microorganisms.

[bib0004] Bankevich A., Nurk S., Antipov D., Gurevich A.A., Dvorkin M., Kulikov A.S., Lesin V.M., Nikolenko S.I., Pham S., Prjibelski A.D., Pyshkin A.V., Sirotkin A.V., Vyahhi N., Tesler G., Alekseyev M.A., Pevzner P.A. (2012). SPAdes: a new genome assembly algorithm and its applications to single-cell sequencing. J. Comput. Biol..

[bib0005] Barata R., Saavedra M.J., Almeida G. (2024). A decade of antimicrobial resistance in human and animal *Campylobacter* spp. Isolates. Antibiotics.

[bib0006] Bonilla-Moreno M., Torrecillas M., Laporte-Amargos J., González-Díaz A., Mussetti A., Tubau F., Gudiol C., Domínguez M.A., Martí S., Rodríguez-Sevilla G., Ardanuy C. (2023). Development of meropenem resistance in a multidrug-resistant *Campylobacter coli* strain causing recurrent bacteremia in a hematological malignancy patient. Antimicrob. Agents Chemother..

[bib0007] Bort B., Martí P., Mormeneo S., Mormeneo M., Iranzo M. (2022). Prevalence and antimicrobial resistance of *Campylobacter* spp. Isolated from broilers throughout the supply chain in Valencia, Spain. Foodborne Pathog. Dis..

[bib0008] Cantero G., Correa-Fiz F., Ronco T., Strube M., Cerdà-Cuéllar M., Pedersen K. (2018). Characterization of *Campylobacter jejuni* and *Campylobacter coli* broiler isolates by whole-genome sequencing. Foodborne Pathog. Dis..

[bib0009] Carattoli A., Zankari E., García-Fernández A., Voldby Larsen M., Lund O., Villa L., Møller Aarestrup F., Hasman H. (2014). *In silico* detection and typing of plasmids using PlasmidFinder and plasmid multilocus sequence typing. Antimicrob. Agents Chemother..

[bib0010] Chen S., Zhou Y., Chen Y., Gu J. (2018). Fastp: an ultra-fast all-in-one FASTQ preprocessor. Bioinformatics.

[bib0011] Cobo-Díaz J.F., González Del Río P., Álvarez-Ordóñez A. (2021). Whole resistome analysis in *Campylobacter jejuni* and *C. coli* genomes available in public repositories. Front. Microbiol..

[bib0012] Connerton I.F., Connerton P.L. (2017). Foodborne Diseases.

[bib0013] Dai L., Sahin O., Grover M., Zhang Q. (2020). New and alternative strategies for the prevention, control, and treatment of antibiotic-resistant *Campylobacter*. Transl. Res..

[bib0014] Dai L., Wu Z., Sahin O., Zhao S., Yu E.W., Zhang Q. (2024). Mutation-based mechanism and evolution of the potent multidrug efflux pump RE-CmeABC in *Campylobacter*. Proc. Natl. Acad. Sci..

[bib0015] De Fátima Rauber Würfel S., Jorge S., De Oliveira N.R., Kremer F.S., Sanchez C.D., Campos V.F., Da Silva Pinto L., Da Silva W.P., Dellagostin O.A (2020). *Campylobacter jejuni* isolated from poultry meat in Brazil: *in silico* analysis and genomic features of two strains with different phenotypes of antimicrobial susceptibility. Mol. Biol. Rep..

[bib0016] Elhadidy M., Miller W.G., Arguello H., Álvarez-Ordóñez A., Dierick K., Botteldoorn N. (2019). Molecular epidemiology and antimicrobial resistance mechanisms of *Campylobacter coli* from diarrhoeal patients and broiler carcasses in Belgium. Transbound. Emerg. Dis..

[bib0017] Engberg J., Aarestrup F.M., Taylor D.E., Gerner-Smidt P., Nachamkin I. (2001). Quinolone and macrolide resistance in *Campylobacter jejuni* and *C. coli*: resistance mechanisms and trends in human isolates. Emerg. Infect. Dis..

[bib0018] European Centre for Disease Prevention and Control (ECDC), European Food Safety Authority (EFSA), & European Medicines Agency (EMA) (2024). Antimicrobial consumption and resistance in bacteria from humans and food-producing animals. EFSA J..

[bib0019] European Food Safety Authority (EFSA) (2010). Analysis of the baseline survey on the prevalence of *Campylobacter* in broiler batches and of *Campylobacter* and *Salmonella* on broiler carcasses in the EU, 2008 - part A: *campylobacter* and *Salmonella* prevalence estimates. EFSA J..

[bib0020] European Food Safety Authority (EFSA) & European Centre for Disease Prevention and Control (ECDC) (2023). The european union summary report on antimicrobial resistance in zoonotic and indicator bacteria from humans, animals and food in 2020/2021. EFSA J..

[bib0021] European Food Safety Authority (EFSA) & European Centre for Disease Prevention and Control (ECDC) (2025). The European Union one Health 2024 zoonoses report. EFSA J..

[bib0022] European Food Safety Authority (EFSA) & European Centre for Disease Prevention and Control (ECDC) (2025). The European Union summary report on antimicrobial resistance in zoonotic and indicator bacteria from humans, animals and food in 2022–2023. EFSA J..

[bib0023] Feldgarden M., Brover V., Gonzalez-Escalona N., Frye J.G., Haendiges J., Haft D.H., Hoffmann M., Pettengill J.B., Prasad A.B., Tillman G.E., Tyson G.H., Klimke W. (2021). AMRFinderPlus and the reference gene catalog facilitate examination of the genomic links among antimicrobial resistance, stress response, and virulence. Sci. Rep..

[bib0024] Florez-Cuadrado D., Ugarte-Ruiz M., Quesada A., Palomo G., Domínguez L., Porrero M.C. (2016). Description of an *erm* (B)-carrying *Campylobacter coli* isolate in Europe. J. Antimicrob. Chemother..

[bib0025] García-Fernández A., Artuso I., Marotta F., Di Romualdo R., Arena S., De Marchis M.L., Pitti M., Primavilla S., Napoleoni M., Aschbacher R., Bracco S., Gradassi M., Janowicz A., Garofolo G., Villa L. (2025). WGS-based surveillance for *Campylobacter* spp. in human infections and chicken meat production in Italy (2023). BMC Microbiol..

[bib0026] García-Fernández A., Janowicz A., Marotta F., Napoleoni M., Arena S., Primavilla S., Pitti M., Romantini R., Tomei F., Garofolo G., Villa L. (2024). Antibiotic resistance, plasmids, and virulence-associated markers in human strains of *Campylobacter jejuni* and *Campylobacter coli* isolated in Italy. Front. Microbiol..

[bib0027] Griggs D.J., Peake L., Johnson M.M., Ghori S., Mott A., Piddock L.J.V. (2009). β-lactamase-mediated β-lactam resistance in *Campylobacter* species: prevalence of Cj0299 (*bla*_OXA-61_) and evidence for a novel β-lactamase in *C. jejuni*. Antimicrob. Agents Chemother..

[bib0028] Gupta S.K., Padmanabhan B.R., Diene S.M., Lopez-Rojas R., Kempf M., Landraud L., Rolain J.-M. (2014). ARG-ANNOT, a new bioinformatic tool to discover antibiotic resistance genes in bacterial genomes. Antimicrob. Agents Chemother..

[bib0029] Gurevich A., Saveliev V., Vyahhi N., Tesler G. (2013). QUAST: quality assessment tool for genome assemblies. Bioinformatics.

[bib0030] Havelaar A.H., Kirk M.D., Torgerson P.R., Gibb H.J., Hald T., Lake R.J., Praet N., Bellinger D.C., De Silva N.R., Gargouri N., Speybroeck N., Cawthorne A., Mathers C., Stein C., Angulo F.J., Devleesschauwer B., on behalf of World Health Organization Foodborne Disease Burden Epidemiology Reference Group (2015). World health organization global estimates and regional comparisons of the burden of foodborne disease in 2010. PLOS Med..

[bib0031] He Y., Dykes G.E., Kanrar S., Liu Y., Gunther N.W., Counihan K.L., Lee J., Capobianco J.A. (2025). Comparative genomic analysis of *Campylobacter* plasmids identified in food isolates. Microorganisms.

[bib0032] Hunt M., Silva N.D., Otto T.D., Parkhill J., Keane J.A., Harris S.R. (2015). Circlator: automated circularization of genome assemblies using long sequencing reads. Genome Biol..

[bib0033] Jehanne Q., Bénéjat L., Ducournau A., Aptel J., Pivard M., Gillet L., Jauvain M., Lehours P. (2025). Increasing rates of *erm(B)* and *erm(N)* in human *Campylobacter coli* and *Campylobacter jejuni* erythromycin-resistant isolates between 2018 and 2023 in France. Antimicrob. Agents Chemother..

[bib0034] Jehanne Q., Bénéjat L., Ducournau A., Domingues-Martins C., Cousinou T., Bessède E., Lehours P. (2021). Emergence of erythromycin resistance methyltransferases in *Campylobacter coli* strains in France. Antimicrob. Agents Chemother..

[bib0035] Jolley K.A., Bray J.E., Maiden M.C.J (2018). Open-access bacterial population genomics: BIGSdb software, the PubMLST.Org website and their applications. Wellcome Open Res..

[bib0036] Jones A.K., Rigby D., Burton M., Millman C., Williams N.J., Jones T.R., Wigley P., O’Brien S.J., Cross P., for the ENIGMA Consortium (2016). Restaurant cooking trends and increased risk for *Campylobacter* infection. Emerg. Infect. Dis..

[bib0037] Karki A.B., Wells H., Fakhr M.K. (2019). Retail liver juices enhance the survivability of *Campylobacter jejuni* and *Campylobacter coli* at low temperatures. Sci. Rep..

[bib0038] Klena J.D., Parker C.T., Knibb K., Ibbitt J.C., Devane P.M.L., Horn S.T., Miller W.G., Konkel M.E. (2004). Differentiation of *Campylobacter coli, Campylobacter jejuni, Campylobacter lari*, and *Campylobacter upsaliensis* by a multiplex PCR developed from the nucleotide sequence of the lipid a gene *lpxA*. J. Clin. Microbiol..

[bib0039] Lakin S.M., Dean C., Noyes N.R., Dettenwanger A., Ross A.S., Doster E., Rovira P., Abdo Z., Jones K.L., Ruiz J., Belk K.E., Morley P.S., Boucher C. (2017). MEGARes: an antimicrobial resistance database for high throughput sequencing. Nucleic Acids Res..

[bib0041] Luo N., Pereira S., Sahin O., Lin J., Huang S., Michel L., Zhang Q. (2005). Enhanced *in vivo* fitness of fluoroquinolone-resistant *Campylobacter jejuni* in the absence of antibiotic selection pressure. Proc. Natl. Acad. Sci..

[bib0042] Manzanares-Pedrosa A., Correa-Fiz F., Andrade F., Ayats T., Nofrarías M., Cerdà-Cuéllar M. (2025). Phenotypic and whole genome-based characterization of antibiotic resistance of *Campylobacter jejuni* isolates from chicken livers. Poult. Sci..

[bib0043] Manzanares-Pedrosa A., Szumilas J., Ayats T., Nofrarías M., Cerdà-Cuéllar M. (2025). *Campylobacter jejuni* and *Campylobacter coli* in broiler chicken livers: high prevalence and surface contamination, but low load in inner tissue. Poult. Sci..

[bib0044] Marin C., Sevilla-Navarro S., Lonjedo R., Catalá-Gregori P., Ferrús M.A., Vega S., Jiménez-Belenguer A. (2020). Genotyping and molecular characterization of antimicrobial resistance in thermophilic *Campylobacter* isolated from poultry breeders and their progeny in Eastern Spain. Poult. Sci..

[bib0045] Marotta F., Garofolo G., Di Marcantonio L., Di Serafino G., Neri D., Romantini R., Sacchini L., Alessiani A., Di Donato G., Nuvoloni R., Janowicz A., Di Giannatale E. (2019). Antimicrobial resistance genotypes and phenotypes of *Campylobacter jejuni* isolated in Italy from humans, birds from wild and urban habitats, and poultry. PLoS. One.

[bib0046] Maurille C., Guérin F., Jehanne Q., Audemard-Verger A., Isnard C., Verdon R., Lehours P., Bonnet R., Giard J.-C., Le Hello S., Gravey F. (2024). Occurrence of *in vivo* carbapenem-resistant *Campylobacter coli* mediated by *porA* point mutation and overexpression of blaOXA-489 under meropenem treatment. Clin. Microbiol. Infect..

[bib0047] McArthur A.G., Waglechner N., Nizam F., Yan A., Azad M.A., Baylay A.J., Bhullar K., Canova M.J., De Pascale G., Ejim L., Kalan L., King A.M., Koteva K., Morar M., Mulvey M.R., O’Brien J.S., Pawlowski A.C., Piddock L.J.V., Spanogiannopoulos P., Wright G.D. (2013). The comprehensive antibiotic resistance database. Antimicrob. Agents Chemother..

[bib0048] Melero B., Juntunen P., Hänninen M.-L., Jaime I., Rovira J. (2012). Tracing *Campylobacter jejuni* strains along the poultry meat production chain from farm to retail by pulsed-field gel electrophoresis, and the antimicrobial resistance of isolates. Food Microbiol..

[bib0049] Moore J.E., Barton M.D., Blair I.S., Corcoran D., Dooley J.S.G., Fanning S., Kempf I., Lastovica A.J., Lowery C.J., Matsuda M., McDowell D.A., McMahon A., Millar B.C., Rao J.R., Rooney P.J., Seal B.S., Snelling W.J., Tolba O. (2006). The epidemiology of antibiotic resistance in *Campylobacter*. Microbes Infect..

[bib0050] Nafarrate I., Lasagabaster A., Sevillano E., Mateo E. (2021). Prevalence, molecular typing and antimicrobial susceptibility of *Campylobacter* spp. Isolates in northern Spain. J. Appl. Microbiol..

[bib0051] National Plan against Antibiotic Resistance (PRAN). (2019). Spain reduces antibiotic consumption by 72% in human health and veterinary antibiotic sales by 32.4%*.* https://www.resistenciaantibioticos.es/es/noticias/espana-reduce-un-72-el-consumo-de-antibioticos-en-salud-humana-y-un-324-las-ventas-de?utm.

[bib0052] Noormohamed A., Fakhr M.K. (2012). Incidence and antimicrobial resistance profiling of *Campylobacter* in retail chicken livers and gizzards. Foodborne Pathog. Dis..

[bib0053] Nunes A., Oleastro M., Alves F., Liassine N., Lowe D.M., Benejat L., Ducounau A., Jehanne Q., Borges V., Gomes J.P., Godbole G., Philippe L. (2023). Recurrent *Campylobacter jejuni* infections with *in vivo* selection of resistance to macrolides and carbapenems: molecular characterization of resistance determinants. Microbiol. Spectr..

[bib0054] Ocejo M., Oporto B., Lavín J.L., Hurtado A. (2021). Whole genome-based characterisation of antimicrobial resistance and genetic diversity in *Campylobacter jejuni* and *Campylobacter coli* from ruminants. Sci. Rep..

[bib0055] Okello W., Nanteza A., Opiyo F., Okello J., Ninsiima L.R., Marin P., Onafruo D., Pithua P., Kankya C., Odoch T. (2023). Detection of carbapenem resistance genes in *Campylobacter col*i and *Campylobacter jejuni* isolated from chickens, and diarrheic children aged less than five years in Kampala city, Uganda. Infect. Dis..

[bib0056] Ortega-Sanz I., Barbero-Aparicio J.A., Canepa-Oneto A., Rovira J., Melero B. (2023). CamPype: an open-source workflow for automated bacterial whole-genome sequencing analysis focused on *Campylobacter*. BMC Bioinform..

[bib0057] Pérez-Boto D., Herrera-León S., García-Peña F.J., Abad-Moreno J.C., Echeita M.A. (2014). Molecular mechanisms of quinolone, macrolide, and tetracycline resistance among *Campylobacter* isolates from initial stages of broiler production. Avian Pathol..

[bib0058] Prendergast D.M., O’Keeffe R., Johnston D., McLernon J., Power F., Byrne B., Gutierrez M. (2025). Prevalence and molecular characterization of *Campylobacter* spp. Isolated from chicken, beef, pork and sheep livers at Irish abattoirs. Int. J. Food Microbiol..

[bib0059] Qin S., Wang Y., Zhang Q., Chen X., Shen Z., Deng F., Wu C., Shen J. (2012). Identification of a novel genomic island conferring resistance to multiple aminoglycoside antibiotics in *Campylobacter coli*. Antimicrob. Agents Chemother..

[bib0060] Qin S., Wang Y., Zhang Q., Zhang M., Deng F., Shen Z., Wu C., Wang S., Zhang J., Shen J. (2014). Report of ribosomal RNA methylase gene *erm(B)* in multidrug-resistant *Campylobacter coli*. J. Antimicrob. Chemother..

[bib0061] Shen Z., Wang Y., Zhang Q., Shen J. (2018). Antimicrobial resistance in *Campylobacter* spp. Microbiol. Spectr..

[bib0062] Signorini M.L., Rossler E., Díaz David D.C., Olivero C.R., Romero-Scharpen A., Soto L.P., Astesana D.M., Berisvil A.P., Zimmermann J.A., Fusari M.L., Frizzo L.S., Zbrun M.V. (2018). Antimicrobial resistance of thermotolerant *Campylobacter* species isolated from humans, food-producing animals, and products of animal origin: a worldwide meta-analysis. Microb. Drug Resist..

[bib0063] Simaluiza R., Toledo Z., Ochoa S., Fernandez H. (2015). The prevalence and antimicrobial resistance of *Campylobacter jejuni* and *Campylobacter coli* in chicken livers used for human consumption in Ecuador. J. Anim. Vet. Adv..

[bib0064] Sommer H.M., Høg B.B., Larsen L.S., Sørensen A.I.V., Williams N., Merga J.Y., Cerdà-Cuéllar M., Urdaneta S., Dolz R., Wieczorek K., Osek J., David B., Hofshagen M., Jonsson M., Wagenaar J.A., Bolder N., Rosenquist H. (2016). Analysis of farm specific risk factors for *Campylobacter* colonization of broilers in six European countries. Microb. Risk Anal..

[bib0065] Varga C., Guerin M.T., Brash M.L., Slavic D., Boerlin P., Susta L. (2019). Antimicrobial resistance in *Campylobacter jejuni* and *Campylobacter coli* isolated from small poultry flocks in Ontario, Canada: a two-year surveillance study. PLoS One.

[bib0066] Warburton P.J., Amodeo N., Roberts A.P. (2016). Mosaic tetracycline resistance genes encoding ribosomal protection proteins. J. Antimicrob. Chemother..

[bib0067] Whitehouse C.A., Young S., Li C., Hsu C.-H., Martin G., Zhao S. (2018). Use of whole-genome sequencing for *Campylobacter* surveillance from NARMS retail poultry in the United States in 2015. Food Microbiol..

[bib0068] Wick R.R., Judd L.M., Gorrie C.L., Holt K.E. (2017). Unicycler: resolving bacterial genome assemblies from short and long sequencing reads. PLOS Comput. Biol..

[bib0069] Wieczorek K., Osek J. (2013). Antimicrobial resistance mechanisms among *Campylobacter*. BioMed Res. Int..

[bib0070] Wieczorek K., Wołkowicz T., Osek J. (2018). Antimicrobial resistance and virulence-associated traits of *Campylobacter jejuni* isolated from poultry food chain and humans with diarrhea. Front. Microbiol..

[bib0071] World Health Organization (WHO). (2019). WHO List of medically important antimicrobials. https://Doi.org/ISBN/2520978-92-4-008461-2.

[bib0072] World Health Organization. (2024). WHO LIST OF MEDICALLY IMPORTANT ANTIMICROBIAls. https://cdn.who.int/media/docs/default-source/gcp/who-mia-list-2024-lv.pdf.

[bib0073] Yang M., Wang X., Zheng L., Zhu Y. (2025). Genomic analysis and antimicrobial resistance in human- and poultry-derived *Campylobacter jejuni* isolates from Hangzhou, China. Front. Microbiol..

[bib0074] Yao H., Jiao D., Zhao W., Li A., Li R., Du X.-D. (2020). Emergence of a novel *tet*(L) variant in *Campylobacter* spp. Of chicken origin in China. Antimicrob. Agents Chemother..

[bib0075] Zankari E., Hasman H., Cosentino S., Vestergaard M., Rasmussen S., Lund O., Aarestrup F.M., Larsen M.V. (2012). Identification of acquired antimicrobial resistance genes. J. Antimicrob. Chemother..

[bib0076] Zarske M., Luu H.Q., Deneke C., Knüver M.-T., Thieck M., Hoang H.T.T., Bretschneider N., Pham N.T., Huber I., Stingl K. (2024). Identification of knowledge gaps in whole-genome sequence analysis of multi-resistant thermotolerant *Campylobacter* spp. BMC Genom..

[bib0077] Zhang P., Zhang X., Liu Y., Cui Q., Qin X., Niu Y., Wang C., Wang T., Chen Q., Ding S., Ma X., Shen Z. (2022). Genomic insights into the increased occurrence of campylobacteriosis caused by antimicrobial-resistant *Campylobacter coli*. mBio.

[bib0078] Zhang X., Zhou Q., Tang M., Pu J., Zhang J., Lu J., Zhang Y., Gao Y. (2021). Aminoglycoside resistance and possible mechanisms in *Campylobacter* spp. Isolated from chicken and swine in Jiangsu, China. Front. Microbiol..

[bib0079] Zhuo R., Younes R.L., Ward K., Yang S. (2024). Carbapenem resistant *Campylobacter jejuni* bacteremia in a Bruton’s X-linked agammaglobulinemia patient. Eur. J. Clin. Microbiol. Infect. Dis..

