# Supplementary material for: Phenotypic and whole genome characterization of multidrug-resistant Campylobacter coli from chicken liver
Source: Poult Sci. 2026 Jun 10;105(10):107271. doi: 10.1016/j.psj.2026.107271 (PMC13324448; doi:10.1016/j.psj.2026.107271)
Supplement: Supplementary file 1 [file mmc1.docx]

Supplementary table 1. Assembly metrics. N50, minimum contig length at which 50 % of the total assembly is reached.

| Isolates | N50 | Assembly size (bp) | No. contigs | GC (%) |
| --- | --- | --- | --- | --- |
| 1 | 218253 | 1755047 | 31 | 31,13 |
| 2 | 162722 | 1737825 | 23 | 31,36 |
| 3 | 179370 | 1755154 | 36 | 31,14 |
| 4 | 268604 | 1682629 | 24 | 31,45 |
| 5 | 172672 | 1668510 | 13 | 31,39 |
| 6 | 190573 | 1788971 | 46 | 31,1 |
| 7 | 217980 | 1784479 | 48 | 31,14 |
| 8 | 285102 | 1852648 | 17 | 31,15 |
| 9 | 173797 | 1751571 | 35 | 31,21 |
| 10 | 218074 | 1675220 | 21 | 31,37 |
| 11 | 216185 | 1767072 | 33 | 31,15 |
| 12 | 191142 | 1820528 | 40 | 30,93 |
| 13 | 223977 | 1704980 | 18 | 31,39 |
| 14 | 255800 | 1739626 | 14 | 31,26 |
| 15 | 238526 | 1823603 | 43 | 31,04 |
| 16 | 190167 | 1916457 | 49 | 30,91 |
| 17 | 265084 | 1696744 | 11 | 31,35 |
| 18 | 256068 | 1639939 | 24 | 31,39 |
| 19 | 279827 | 1736285 | 17 | 31,26 |
| 20 | 218515 | 1686262 | 16 | 31,3 |
| 21 | 261541 | 1892376 | 43 | 31,11 |
| 22 | 286055 | 1713741 | 9 | 31,5 |
| 23 | 273201 | 1670716 | 18 | 31,48 |
| 24 | 279827 | 1736158 | 16 | 31,26 |
| 25 | 273198 | 1658755 | 12 | 31,42 |
| 26 | 273199 | 1658620 | 13 | 31,42 |
| 27 | 162522 | 1777843 | 59 | 31,28 |
| 28 | 218659 | 1687032 | 17 | 31,3 |
| 29 | 218479 | 1687034 | 17 | 31,3 |
| 30 | 273021 | 1686653 | 11 | 31,35 |
| 31 | 238176 | 1706648 | 15 | 31,35 |
| 32 | 184154 | 1712457 | 68 | 31,43 |
| 33 | 173968 | 1745955 | 35 | 31,14 |
| 34 | 261541 | 1893664 | 49 | 31,12 |
| 35 | 265042 | 1732940 | 22 | 31,29 |
| 36 | 162685 | 1835129 | 19 | 31,15 |
| 37 | 241867 | 1741961 | 27 | 31,41 |
| 38 | 286055 | 1740540 | 50 | 31,57 |
| 39 | 220381 | 1659549 | 24 | 31,38 |
| 40 | 174795 | 1813561 | 92 | 31,07 |

Supplementary table 2. Point mutations identified in the *gyrA* gene of *Campylobacter coli* isolates of unknown role in quinolone resistance.

| Quinolone unknown point mutations | | | | | | | | | | | |
| --- | --- | --- | --- | --- | --- | --- | --- | --- | --- | --- | --- |
| Isolates | Cip^a^ | S22G | V149I | A206T | T269I | R285K | N404S | L483P | A664V | T665S | F723L |
| 1 | R(2) | − | − | − | − | − | − | − | − | − | − |
| 2 | R (32) | − | − | − | − | − | − | − | − | − | − |
| 3 | R (8) | − | − | − | − | − | − | − | − | − | − |
| 4 | R (16) | − | − | − | − | − | − | − | − | − | − |
| 5 | R (8) | − | − | GCT>ACT; A>T ^b^ | − | − | − | − | − | − | − |
| 6 | R (16) | − | − | − | − | − | − | − | − | − | − |
| 7 | R (16) | − | − | GCT>ACT; A>T | − | − | − | − | − | − | − |
| 8 | R (32) | − | − | − | − | − | − | − | − | − | − |
| 9 | R (8) | − | − | − | − | − | − | − | − | − | − |
| 10 | R (16) | − | GTT>ATT; V>I | − | − | − | − | − | − | − | − |
| 11 | R (16) | − | − | − | − | − | − | − | − | − | − |
| 12 | R (16) | − | − | − | − | − | − | − | − | − | − |
| 13 | R (8) | − | − | − | − | − | − | − | − | − | − |
| 14 | R(4) | − | − | − | − | − | − | − | − | − | − |
| 15 | R (8) | − | − | GCT>ACT; A>T | − | − | − | − | − | − | − |
| 16 | R (8) | − | − | − | − | − | − | − | − | − | − |
| 17 | R (16) | − | − | − | − | − | AAT>AGT: N>S | − | − | − | TTT>CTT: F>L |
| 18 | R (>32) | − | − | − | − | − | − | − | − | − | − |
| 19 | R (32) | − | − | GCT>ACT; A>T | − | − | − | − | − | − | − |
| 20 | R (8) | − | − | − | − | − | − | − | − | − | − |
| 21 | R (16) | − | − | − | − | − | − | − | − | − | − |
| 22 | R (8) | − | − | − | − | − | − | − | − | − | − |
| 23 | R (32) | − | − | − | − | − | − | − | − | − | − |
| 24 | R (16) | − | − | GCT>ACT; A>T | − | − | − | − | − | − | − |
| 25 | R (8) | − | − | − | − | − | − | − | − | − | − |
| 26 | R(4) | − | − | − | − | − | − | − | − | − | − |
| 27 | R (16) | − | − | − | − | − | − | − | − | − | − |
| 28 | R (8) | − | − | − | − | − | − | − | − | − | − |
| 29 | R (16) | − | − | − | − | − | − | − | − | ACT>AGT; T>S | − |
| 30 | R (16) | − | − | − | − | − | − | − | − | − | − |
| 31 | R (16) | AGT>GGT; S>G | − | − | ACC>ATC; T>I | AGG>AAG; R>K | − | − | GCC>GTT; A>V | − | − |
| 32 | R (16) | − | − | − | − | − | − | − | − | − | − |
| 33 | R (8) | − | − | − | − | − | − | − | − | − | − |
| 34 | R (16) | − | − | − | − | − | − | − | − | − | − |
| 35 | R (8) | − | − | − | − | − | − | − | − | − | − |
| 36 | R (16) | − | − | − | − | − | − | CCG>CTG; P>L | − | − | − |
| 37 | R (16) | − | − | − | − | − | − | − | − | − | − |
| 38 | R (16) | − | − | − | − | − | − | − | − | − | − |
| 39 | R (16) | − | − | − | − | − | − | − | − | − | − |
| 40 | R (32) | − | − | − | − | − | − | − | − | − | − |

^a^ Cip, ciprofloxacin; Interpretation of MIC values for *C. coli* epidemiological cutoff value: R > 0,5 mg/L.

^b^ Codon changes; amino acid changes: A, alanine; F, phenylalanine; G, glycine; I, isoleucine; K, lysine; L, leucine; N, asparagine; P, proline; R, arginine; S, serine; T, threonine; V, valine.

Supplementary Table 3. Point mutations identified in the 23S rDNA gene of *Campylobacter coli* isolates, not previously associated with erythromycin resistance.

| Macrolide unknown mutations | | | | | | | | | | | | | | | | | | |
| --- | --- | --- | --- | --- | --- | --- | --- | --- | --- | --- | --- | --- | --- | --- | --- | --- | --- | --- |
| Isolates | Ery | C240T | C296G | G327A | G364C | G402A | T416G | C418T | A554C | T571G | G1185T | C1190T | T1191C | T1219C | A1496G | G1579A | A1730C | T1735C |
| 1 | R(64) | + | + | − | − | + | + | − | + | + | − | − | − | − | − | − | + | + |
| 2 | S(≤1) | − | − | − | − | − | − | − | − | − | − | − | − | − | − | − | − | − |
| 3 | S(≤1) | − | − | − | − | − | − | − | − | − | − | − | − | − | − | − | − | − |
| 4 | R(512) | + | + | − | + | + | + | + | + | + | − | − | − | + | − | + | + | + |
| 5 | S(≤1) | − | − | − | − | − | − | − | − | − | − | − | − | − | − | − | − | − |
| 6 | R(>512) | + | + | − | + | + | + | + | + | + | − | − | − | − | − | − | + | + |
| 7 | R(512) | + | + | − | + | + | + | + | + | + | − | − | − | − | − | − | + | + |
| 8 | S(≤1) | − | − | − | − | − | − | − | − | − | − | − | − | − | − | − | − | − |
| 9 | R(>512) | + | + | − | + | + | + | + | + | + | − | − | − | − | − | − | + | + |
| 10 | R(>512) | − | − | − | − | − | − | − | − | − | − | − | − | − | − | − | − | − |
| 11 | R(>512) | − | + | − | + | + | + | + | + | + | − | − | − | − | − | − | + | + |
| 12 | R(>512) | + | + | − | + | + | + | − | + | − | − | − | − | − | − | − | + | + |
| 13 | R(512) | + | + | − | + | + | + | + | + | + | − | − | − | − | − | − | + | + |
| 14 | R(64) | − | − | + | + | − | − | − | + | + | − | − | − | − | + | − | + | + |
| 15 | R(>512) | + | + | − | + | + | + | + | + | + | − | − | − | − | − | − | + | + |
| 16 | S(≤1) | − | − | − | − | − | − | − | − | − | − | − | − | − | − | − | − | − |
| 17 | S(≤1) | − | − | − | − | − | − | − | − | − | − | − | − | − | − | − | − | − |
| 18 | R(>512) | − | + | − | + | + | + | + | + | + | − | − | − | − | − | − | + | + |
| 19 | R(>512) | − | + | − | + | + | + | + | + | + | − | − | − | + | − | − | + | + |
| 20 | S(≤1) | − | − | − | − | − | − | − | − | − | − | − | − | − | − | − | − | − |
| 21 | R(>512) | − | + | − | + | + | + | + | + | + | − | − | − | − | − | + | + | + |
| 22 | S(≤1) | − | − | − | − | − | − | − | − | − | − | − | − | − | − | − | − | − |
| 23 | R(64) | − | − | − | − | − | − | − | − | − | − | − | − | − | − | − | − | − |
| 24 | S(≤1) | − | + | − | + | + | + | + | + | + | − | − | − | + | − | − | + | + |
| 25 | R(32) | − | − | − | − | − | − | − | − | − | − | − | − | − | − | − | − | − |
| 26 | R(32) | − | − | − | − | − | − | − | − | − | − | − | − | − | − | − | − | − |
| 27 | S(≤1) | − | + | − | + | + | + | + | + | + | − | − | − | − | − | − | + | + |
| 28 | S(2) | − | − | − | − | − | − | − | − | − | − | − | − | − | − | − | − | − |
| 29 | S(2) | − | − | − | − | − | − | − | − | − | − | − | − | − | − | − | − | − |
| 30 | S(2) | − | − | − | − | − | − | − | − | − | − | − | − | − | − | − | − | − |
| 31 | S(≤1) | − | − | − | − | − | − | − | − | − | − | − | − | − | − | − | − | − |
| 32 | R(>512) | − | + | − | + | + | + | + | + | + | + | + | + | + | − | − | + | + |
| 33 | S(≤1) | − | − | − | − | − | − | − | − | − | − | − | − | − | − | − | − | − |
| 34 | R(512) | − | + | − | + | + | + | + | + | + | − | − | − | − | − | + | + | + |
| 35 | S(≤1) | − | − | − | − | − | − | − | − | − | − | − | − | − | − | − | − | − |
| 36 | R(>512) | − | + | − | + | + | + | + | + | + | − | − | − | − | − | − | + | + |
| 37 | R(>512) | − | + | − | + | + | + | + | + | + | − | − | − | − | − | − | + | + |
| 38 | S(2) | − | − | − | − | − | − | − | − | − | − | − | − | − | − | − | − | − |
| 39 | S(≤1) | − | − | − | − | − | − | − | − | − | − | − | − | − | − | − | − | − |
| 40 | S(≤1) | − | + | − | + | + | + | + | + | + | − | − | − | − | − | − | + | + |

| Macrolide unknown mutations | | | | | | | | | | | | | | | | | | |
| --- | --- | --- | --- | --- | --- | --- | --- | --- | --- | --- | --- | --- | --- | --- | --- | --- | --- | --- |
| Isolates | Ery^a^ | T1752C | A1759G | G1761A | C2097T | C2113T | G2122A | A2123G | G2151A | T2152G | A2153C | T2156G | G2167C | T2172A | C2199T | C2366T | T2544C | A2658G |
| 1 | R(64) | + | + | + | − | − | − | − | − | − | − | − | − | − | − | − | − | − |
| 2 | S(≤1) | − | − | − | − | − | − | − | − | − | − | − | − | − | − | − | − | − |
| 3 | S(≤1) | − | − | − | − | − | − | − | − | − | − | − | − | − | − | − | − | − |
| 4 | R(512) | + | + | + | − | + | − | − | − | − | − | − | − | − | − | − | − | − |
| 5 | S(≤1) | − | − | − | − | − | − | − | − | − | − | − | − | − | − | − | − | − |
| 6 | R(>512) | + | + | − | − | + | − | − | − | − | − | − | − | − | − | − | − | − |
| 7 | R(512) | + | + | + | − | + | − | − | − | − | − | − | − | − | − | − | − | − |
| 8 | S(≤1) | − | − | − | − | − | − | − | − | − | − | − | − | − | − | − | − | − |
| 9 | R(>512) | + | + | + | − | + | − | − | − | − | − | − | − | − | − | − | − | − |
| 10 | R(>512) | − | − | − | − | − | − | − | − | − | − | − | − | − | − | − | − | − |
| 11 | R(>512) | + | + | + | − | + | − | − | − | − | − | − | − | − | − | − | − | − |
| 12 | R(>512) | + | + | + | − | + | − | − | − | − | − | − | − | − | − | − | − | − |
| 13 | R(512) | + | + | + | − | + | − | − | − | − | − | − | − | − | − | − | − | − |
| 14 | R(64) | + | + | + | − | − | − | − | − | − | − | − | − | − | − | + | − | − |
| 15 | R(>512) | + | + | + | − | + | − | − | − | − | − | − | − | − | − | − | − | − |
| 16 | S(≤1) | − | − | − | − | − | − | − | − | − | − | − | − | − | − | − | − | − |
| 17 | S(≤1) | − | − | − | − | − | − | − | − | − | − | − | − | − | − | − | − | − |
| 18 | R(>512) | + | + | + | − | + | − | − | − | − | − | − | − | − | − | − | − | − |
| 19 | R(>512) | + | + | + | − | + | − | − | − | − | − | − | − | − | − | − | − | − |
| 20 | S(≤1) | − | − | − | − | − | − | − | − | − | − | − | − | − | − | − | − | − |
| 21 | R(>512) | + | + | + | − | + | − | − | − | − | − | − | − | − | − | − | − | − |
| 22 | S(≤1) | − | − | − | − | − | − | − | − | − | − | − | − | − | − | − | − | − |
| 23 | R(64) | − | − | − | − | − | − | − | − | − | − | − | − | − | − | − | − | − |
| 24 | S(≤1) | + | + | + | − | + | − | − | − | − | − | − | − | − | − | − | − | − |
| 25 | R(32) | − | − | − | − | − | − | − | − | − | − | − | − | − | − | − | − | − |
| 26 | R(32) | − | − | − | − | − | − | − | − | − | − | − | − | − | − | − | − | − |
| 27 | S(≤1) | + | + | + | − | + | − | − | − | − | − | − | − | − | − | − | − | + |
| 28 | S(2) | − | − | − | − | − | − | − | − | − | − | − | − | − | − | − | − | − |
| 29 | S(2) | − | − | − | − | − | − | − | − | − | − | − | − | − | − | − | − | − |
| 30 | S(2) | − | − | − | − | − | − | − | − | − | − | − | − | − | − | − | − | − |
| 31 | S(≤1) | − | − | − | − | − | − | − | − | − | − | − | − | − | − | − | − | − |
| 32 | R(>512) | + | + | + | − | + | − | − | − | − | − | − | − | − | − | − | − | − |
| 33 | S(≤1) | − | − | − | − | − | − | − | − | − | − | − | − | − | − | − | − | − |
| 34 | R(512) | + | + | + | − | + | − | − | − | − | − | − | − | − | − | − | − | − |
| 35 | S(≤1) | − | − | − | − | − | − | − | − | − | − | − | − | − | − | − | − | − |
| 36 | R(>512) | + | + | + | + | + | + | + | + | + | + | + | + | + | + | − | − | − |
| 37 | R(>512) | + | + | + | − | + | − | − | − | − | − | − | − | − | − | − | + | − |
| 38 | S(2) | − | − | − | − | − | − | − | − | − | − | − | − | − | − | − | − | − |
| 39 | S(≤1) | − | − | − | − | − | − | − | − | − | − | − | − | − | − | − | − | − |
| 40 | S(≤1) | + | + | + | − | + | − | − | − | − | − | − | − | − | − | − | − | − |

(Continued)

^a^ Ery, erythromycin; Interpretation of MIC values for *C. coli* epidemiological cutoff values: Ery (R > 8 mg/L).
